# Liquid-Phase Exfoliation of Ta_2_NiS_5_ and Its Application in Near-Infrared Mode-Locked Fiber Lasers with Evanescent Field Interactions and Passively Q-Switched Bulk Laser

**DOI:** 10.3390/nano12040695

**Published:** 2022-02-19

**Authors:** Shunxiang Liu, Hongfu Huang, Jinsheng Lu, Ning Xu, Junle Qu, Qiao Wen

**Affiliations:** Key Laboratory of Optoelectronic Devices and Systems of Ministry of Education and Guangdong Province, College of Physics and Optoelectronic Engineering, Shenzhen University, Shenzhen 518060, China; 2150190124@email.szu.edu.cn (S.L.); 1900453012@email.szu.edu.cn (H.H.); 1910454045@email.szu.edu.cn (J.L.); 1810285032@email.szu.edu.cn (N.X.); jlqu@szu.edu.cn (J.Q.)

**Keywords:** ternary chalcogenide, saturable absorber, Q-switched bulk laser, ultrafast fiber laser

## Abstract

We report on the application of a 1 μm solid-state passively Q-switched (PQS) laser and 1, 1.5 μm mode-locked (ML) fiber lasers based on ternary chalcogenide Ta_2_NiS_5_ saturable absorber (SA), which were successfully fabricated by liquid-phase exfoliation method (LPE). The nonlinear absorption of the Ta_2_NiS_5_-SA was characterized by 0.32 GW/cm^2^ and 0.25 GW/cm^2^ saturation intensities with 7.3% and 5.1% modulations depths at 1 μm and 1.5 μm, respectively. A PQS solid-state laser operating at 1.0 μm has been realized with the Ta_2_NiS_5_-SA. The maximum average output power, shortest pulse width, pulse energy, and pulse peak power from the PQS laser are 0.257 W, 180 ns, 1.265 μJ, and 7 W. Moreover, highly stable femtosecond laser centered at 1.5 μm, and picosecond centered at 1 μm, ML fiber lasers were obtained using the Ta_2_NiS_5_-SA. A 70 dB signal-to-noise ML laser with a pulse duration of 781 fs was observed in the telecommunication window, which is better than the duration of the previously reported lasers based on Ta_2_NiS_5_. The corresponding maximum single pulse energy and peak power are 0.977 nJ and 1251 W, respectively. The Ta_2_NiS_5_-SA fabricated by the LPE method was applied in near-infrared (NIR) ML fiber lasers (evanescent field interactions) and PQS bulk lasers. The results indicate that Ta_2_NiS_5_-SA prepared by the LPE method can be applied in a 1 μm bulk PQS laser and improved by the new combination mode (evanescent field interactions) for better output performance of the fiber laser.

## 1. Introduction

Near-infrared (NIR) pulse lasers have been most commonly applied in the fields of material processing, biomedical research, laser processing, and lidar due to their special wavelength, high peak power, and short pulse width [1,2,3,4]. Passively Q-switched (PQS) and mode-locked (ML) techniques are the main ways to generate pulse lasers, and the saturable absorber (SA) is the key element. Since graphene was first discovered in 2004 [5], the study of two-dimensional (2D) materials has attracted much attention because of their unique structures and excellent photoelectric properties [6,7,8]. These properties endow these materials with tremendous potential in optoelectronic applications. Graphene [9,10,11], black phosphorus (BP) [12,13,14], transition metal dichalcogenides (TMDs, including MoS_2_ [15], WS_2_ [16], NbS_2_ [17], TiS_2_ [18], and SnS_2_ [19]) and topological insulators (TIs, including Bi_2_Te_3_ [20] and Sb_2_Te_3_ [21]) have been used as basic components of photonic devices, including all-optical modulators and optical switches, in ultrafast laser generation. However, some inherent defects of these materials limit their application and further development. For example, graphene has a zero bandgap and a weak electronic switching ratio, resulting in low photon absorption efficiency. BP [22], with a tunable bandgap ranging from 0.3 eV (bulk) to 1.5 eV (monolayer), has broadband saturable absorption characteristics from the visible to mid-infrared region. Regrettably, it is unstable and easily oxidized. Although the high absorption efficiency and optical response of TMDs are satisfactory, the large bandgap limits their application [23]. TIs are electronic materials with a bulk bandgap similar to an ordinary insulator, but they require a complicated preparation process [24]. This limits the efficient use of photonic equipment based on 2D materials. Thus, exploring a new type of SA with superior performance is a long-term goal.

As new members of the 2D material family, ternary chalcogenides are more attractive because of the stoichiometric variation and synergistic effect arising from the third additional element introduced. Due to their novel physical and chemical properties, ternary chalcogenides have been applied in electronics, optoelectronics, and biosensors [25,26,27]. Ta_2_NiS_5_, as a typical example of the ternary chalcogenide family, has an unusual structure that displays 2D characteristics via a layered crystal structure stacked through weak van der Waals interactions and one-dimensional characteristics of a single-layer chain structure [28]. Moreover, the interesting, layered structure possesses considerable in-plane anisotropy, and each sublayer is three atoms thick. The Ni and Ta atoms of the middle sheet are tetrahedrally and octahedrally coordinated with S atoms, forming NiS_4_ and TaS_6_ units, respectively [29]. Bulk Ta_2_NiS_5_ and few-layer Ta_2_NiS_5_ have been certified to be direct bandgap semiconductors with a bandgap of 0.36 eV, which heralds potential applications in photonics [29]. In 2019, Ta_2_NiS_5_ nanosheets were fabricated by the liquid-phase exfoliation (LPE) method and used as an SA in a PQS all-solid-state laser at 1.9 μm by Yan et al. [30]. Compared with mid-infrared 1.9 μm PQS lasers, the NIR 1 μm PQS lasers are more widely used in various kinds of fields [31,32]. However, no reports about Ta_2_NiS_5_ applied in 1 μm PQS solid-state lasers have been presented. Recently, Ma et al. reported the preparation of Ta_2_NiS_5_ nanosheets by mechanical exfoliation method (ME) and achieved pulsed fiber laser based on a Ta_2_NiS_5_ SA by depositing on the fiber connector end facets as a film [33]. To date, there is no research about fiber lasers based on Ta_2_NiS_5_ with evanescent field interactions. There are two ways to apply the SAs into the fiber laser cavity. One way is to deposit the SA material on the fiber connector end facets as a film. The other (evanescent field interactions) is that the material might be deposited on tapered or side-polished fibers [34]. The combination method using side-polished fibers appears to have advantages over the method of depositing materials on the end face of the fiber connector. Due to the interaction with the evanescent field propagating in the fiber cladding, these SA materials will not be exposed to high optical power. In addition, the length of the interaction between light and the SA is on the order of millimeters (instead of nanometers, when the material is deposited on the connector) [35,36,37].

In this paper, we prepared Ta_2_NiS_5_-SA using the liquid-phase exfoliation method. The LPE is a proficient and effective method for fabricating materials. Compared with techniques such as ME, magnetron sputtering (MS), pulsed laser deposition (PLD), chemical vapor transport (CVT), hydrothermal intercalation/exfoliation (HI/E), and chemical vapor deposition (CVD), this method has the advantages of convenience and practicality [38]. The layers of the prepared Ta_2_NiS_5_-SA nanosheets were 19~23, more than the layers of Ta_2_NiS_5_-SA nanosheets fabricated by the ME method (~2 layers) [33], and our saturation intensity is significantly higher than them. The nonlinear absorption of the Ta_2_NiS_5_-SA at 1 μm and 1.5 μm was measured by Z-scan and P-scan measurements, respectively. 1 μm PQS bulk laser and 1, 1.5 μm ML fiber lasers based on Ta_2_NiS_5_-SA were achieved. In a 1 μm PQS bulk laser, the maximum average output power and minimum pulse width are 0.275 W and 180 ns, respectively. For the ML fiber lasers, femtosecond erbium-doped (EDF) and picosecond ytterbium-doped (YDF) ML fiber lasers with evanescent field interactions were achieved using side-polished fibers. A stable ML fiber laser was achieved at 1557 nm with a pulse duration of 781 fs, shorter than the previous record for Ta_2_NiS_5_ [33]. The results indicate that Ta_2_NiS_5_-SA prepared by the LPE method can be applied in a 1 μm bulk PQS laser and improved by the new combination mode (evanescent field interactions) for better output performance of the fiber laser.

## 2. Experimental

### 2.1. Fabrication and Characterization of the Ta_2_NiS_5_-SA

**Figure 1:**
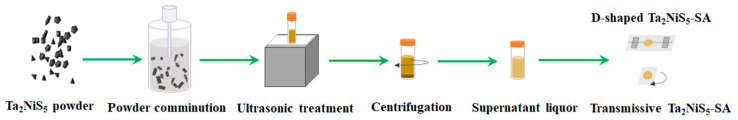
Illustration of the Ta_2_NiS_5_-SA preparation process.

**Figure 2:**
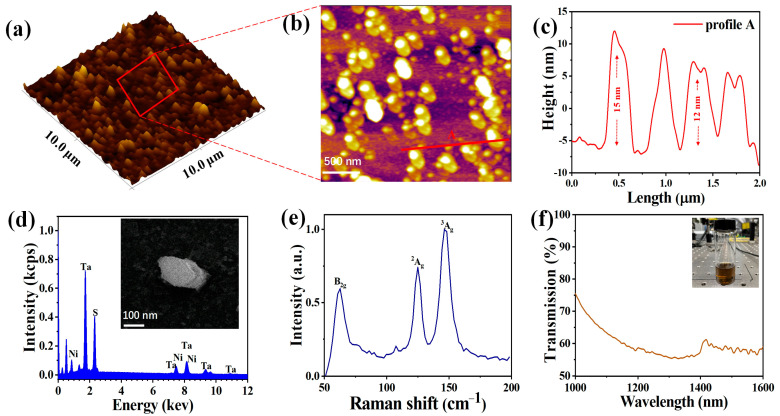
(**a**) 3D Morphology image of Ta_2_NiS_5_ nanosheets. (**b**) 2D Morphology image of Ta_2_NiS_5_ nanosheets. (**c**) Corresponding thickness distribution of Ta_2_NiS_5_ nanosheets. (**d**) EDS image and SEM image (Inset). (**e**) Raman spectrum of the Ta_2_NiS_5_ nanosheet. (**f**) Transmission spectrum of the Ta_2_NiS_5_ nanosheets solution (IPA).

Ternary chalcogenide Ta_2_NiS_5_ powder was purchased from Shenzhen Six Carbon Technology Development Co., Ltd. (Shenzhen, China). The preparation process of the Ta_2_NiS_5_-SA is shown in Figure 1. In detail, 0.3 g Ta_2_NiS_5_ powder was dissolved in 30 mL isopropyl alcohol (IPA, Macklin, Shanghai, China) solution under uniform stirring for 3 h. The dispersion was sonicated at 300 W for 12 h and a temperature below 20 °C. The solution was centrifuged at 6000 rpm for 20 min, and few-layer nanosheets were obtained from the supernatant liquid. Then the supernatant liquid with Ta_2_NiS_5_ nanosheets was spun onto a glass sheet and a (side-polished) D-shaped fiber (drying for 24 h at room temperature) to achieve Ta_2_NiS_5_-SA. 2D materials with different layer numbers have unique optical performances [39]. In the characterization of Ta_2_NiS_5_ nanosheets, the solution material was dropped on the silicon wafer and dried naturally for 24 h firstly to avoid impurity elements from the solution. To investigate the morphology of the fabricated Ta_2_NiS_5_ nanosheets, atomic force microscopy (AFM, MFP-3D Infinity, Asylum Research, Oxford, UK) was used. The 3D and 2D results are shown in Figure 2a,b, the average thickness of the fabricated multi-layer Ta_2_NiS_5_ nanosheets was 12~15 nm throughout the thickness profile as displayed in Figure 2c, corresponding to about 19~23 layers thick (the layer thickness of Ta_2_NiS_5_ is approximately 0.63 nm) [30]. Scanning electron microscopy (SEM, JSM-5910LV, JEOL, Tokyo, Japan) and energy-dispersive X-ray spectroscopy (EDS, Oxford Instruments, Oxford, UK) were used to investigate the micro surface topography and elemental composition of a Ta_2_NiS_5_ nanosheet, where the impurity of C, O, and other elements in the air was excluded, as displayed in Figure 2d. Three elements (S, Ni, Ta) were identified with a weight ratio of 27.29:10.06:62.65, and the corresponding atomic ratio was 62.49:12.38:25.13 (~5:1:2), illustrating the purity of the Ta_2_NiS_5_ nanosheets. Raman measurement of the Ta_2_NiS_5_ nanosheet was conducted by Raman spectroscopy (excitation wavelength: 532 nm, inVia, Renishaw, New Mills, UK), and the result was shown in Figure 2e. Three peaks located at 62.6, 124.9, and 146.2 cm^−^^1^ are observed in the detection range of Figure 2e, corresponding to one twisting motion for mode B_2g_ and two stretching motions for ^2^A_g_ and ^3^A_g_ [29]. Compared with the result in [29], the Raman peaks of the three modes in our results show a little red-shifted and slightly different peak intensities. The first phenomenon results from the thermal anharmonicity of the Ta_2_NiS_5_ nanosheets. The latter occurs mainly due to the thickness-dependent light absorption capacity, optical interference, and band structure with a layer thickness of layer materials [29]. A UV/VIS/NIR spectrophotometer measured the linear transmission spectrum from 1000 nm to 1600 nm was measured by a UV/VIS/NIR spectrophotometer (LAMBDA, Pekin Elmer Inc., Waltham, MA, USA). As shown in Figure 2f, the transmittance of Ta_2_NiS_5_ nanosheets solutions was 73.2%@1036 nm, 72.6%@1064 nm, and 54.2%@1550 nm. All the characterization procedures for Ta_2_NiS_5_ nanosheets solutions were performed at room temperature.

### 2.2. Saturable Absorption Characteristics of the Ta_2_NiS_5_-SA

**Figure 3:**
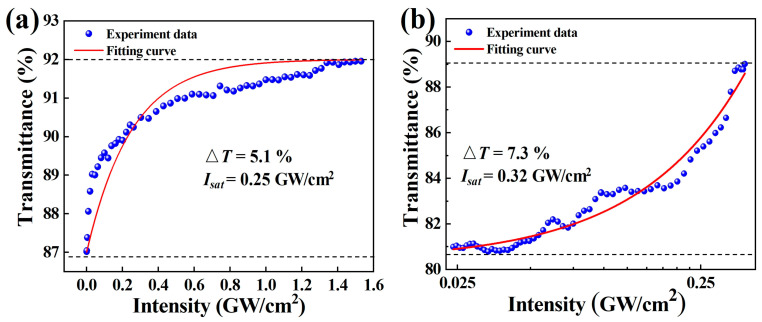
(**a**) Corresponding nonlinear saturable absorption curve in 1.5 μm. (**b**) Corresponding nonlinear saturable absorption curve in 1 μm.

Due to the limitation of experimental conditions, P-scan [34] and Z-scan [40] methods were used to measure the saturable absorption of the Ta_2_NiS_5_-SA. To investigate the nonlinear optical characteristics of the as-fabricated Ta_2_NiS_5_-SA at 1.5 μm, a balanced twin-detector measurement system was employed (1550 nm, 600 fs, 7.36 MHz). Figure 3a shows the P-scan curves of Ta_2_NiS_5_-SA. When only the single-photon absorption is considered, the following formula can be obtained [40,41,42]:

(1) $$T\left(I\right)=1-\Delta T\times exp\left(\frac{-I}{I_{sat}}\right)-T_{ns}$$

where *T*(*I*) is the transmission, ∆*T* is the modulation depth (MD), *I* is the input intensity, *I_sat_* is the saturation intensity, and *T_ns_* is the non-saturable loss (NL).

As shown in Figure 3a, by fitting the curve with the equation, the values of ∆*T*, *I_sat_*, and NL at 1.5 μm were calculated to be ~5.1%, ~0.25 GW/cm^2^, and ~8%, respectively. Besides, the nonlinear optical characteristics of the as-fabricated Ta_2_NiS_5_-SA at 1 μm were measured by an open-aperture Z-scan measuring system (1064 nm, 100 fs, 1 kHz). Figure 3b shows the Z-scan experimental data and fitting curves by the same Formula (1) of Ta_2_NiS_5_-SA. The ∆*T*, *I_sat_*, and NL of the Ta_2_NiS_5_-SA at 1 μm were calculated to be ~7.3%, ~0.32 GW/cm^2^, and ~11.6%, respectively. Compared to the results of the P-scan, the data results of the Z-scan are a little larger. The possible reason is that there are differences in the measurement results of different wavelengths (1 μm, 1.5 μm), and the measurement principles of the P-scan and Z-scan are different. In Z-scan measurement, different incident light intensities are obtained by changing the position of the material behind the lens. Generally, the optical path behind the lens is defined as the z-axis. Besides, the position of the sample is fixed in the P-scan, and the incident light intensity is changed by changing the incident light power to ensure the response of the same sample area under different intensities. All results in either way show the saturable absorption characteristics of the Ta_2_NiS_5_-SA in 1 μm and 1.5 μm.

### 2.3. NIR Solid-State and Fiber Pulse Lasers Based on Ta_2_NiS_5_-SA

**Figure 4:**
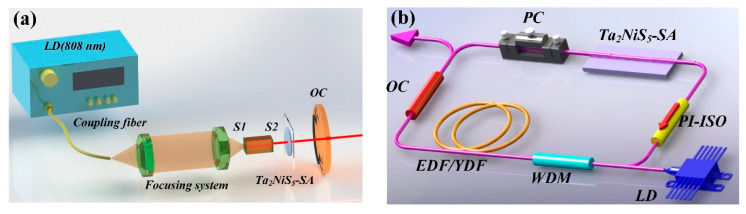
(**a**) Schematic setup of the diode-pumped Ta_2_NiS_5_-SA-based PQS Nd: YAG laser. (**b**) Cavity schematic for the ML fiber laser.

A compact 25 mm plane-concave system was designed to investigate the saturable absorption of Ta_2_NiS_5_-SA applied in an all-solid-state laser, as shown in Figure 4a. The pump source was a commercially available 808 nm diode laser (Dilas, Mainz, Germany) with a coupling fiber (core diameter: 200 μm, NA: 0.22). The pump beam was focused into the laser gain medium by a collimating focusing system (1:0.8) consisting of two lenses. The laser gain medium was a coated 1.2 at% Nd: YAG crystal with a size of 3 × 3 × 4 mm^3^. The laser gain medium (wrapped with indium foils) was embedded in a copper block cooled by circulating water (17 °C) to dissipate the heat. The coated film S1 (HT@808 nm, HR@1064 nm) near the pump side was used as an input mirror; another film S2 (HT@1064 nm, HR@808 nm) was coated to ensure that the gain medium fully absorbs the pump light and protect Ta_2_NiS_5_-SA from the pump light. A concave mirror (partial transmission of 15% at 1064 nm) with a curvature radius of 50 mm was used as an output coupler (OC).

To further research the optical performance of the Ta_2_NiS_5_-SA, two all-fiber lasers were assembled; two fiber laser systems (EDF and YDF) with different operating wavelengths were constructed. A 4 m EDF (4.45 dB/m@980 nm) was used to generate a pulse laser in the NIR telecommunication window (1.5 μm). In addition, a one-meter long YDF (250 dB/m@980 nm) was used to generate a pulse laser at 1 μm. An experimental schematic diagram of the ring cavity design is shown in Figure 4b. The fiber laser system consists of a 980/1550 nm (or 980/1060 nm) wavelength division multiplexer (WDM), a 980 nm laser diode (LD), a polarization-independent isolator (PI-ISO), a polarization controller (PC), an optical coupler (OC), a doped fiber and a D-shaped fiber. The interaction length of the D-shaped fiber is 10 mm, and the distance from the fiber core boundary to the lowest point of the D-shaped region is ~1 μm. The Ta_2_NiS_5_ nanosheets solution was dropped onto the side-polished part of the D-shaped fiber to form SAs inserted between the PC and PI-ISO.

## 3. Results and Discussion

### 3.1. 1 μm PQS Solid-State Nd: YAG Laser-Based on Ta_2_NiS_5_-SA

**Figure 5:**
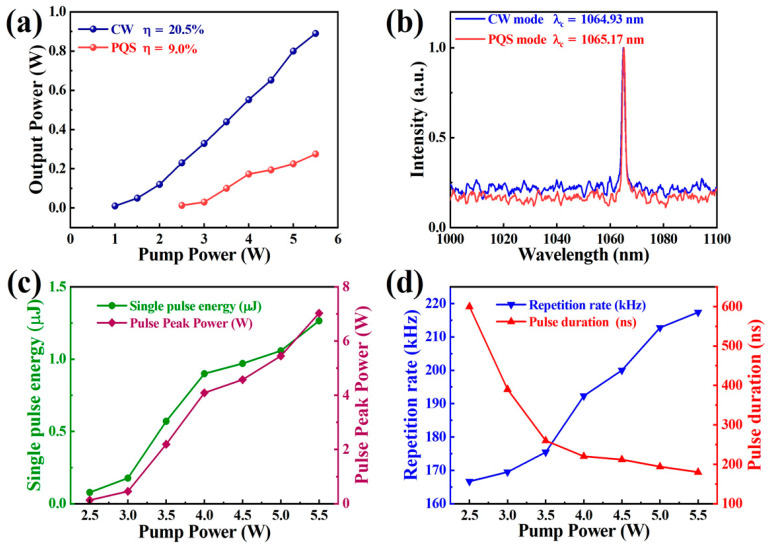
(**a**) Average output power of CW and PQS Nd: YAG lasers versus pump power; (**b**) output spectra of the CW and PQS Nd: YAG lasers; (**c**) single pulse energy and pulse peak power versus pump power; (**d**) pulse repetition rate and pulse duration versus pump power.

**Figure 6:**
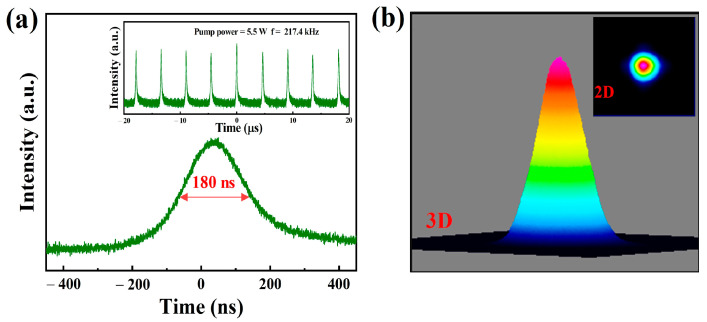
(**a**) Stable pulse train at the highest repetition rate and shortest pulse profile and (**b**) 2D, 3D beam profiles of the PQS Nd: YAG laser at the highest pump power (5.5 W).

CW laser operation was first investigated before carrying out the PQS laser experiment. As shown in Figure 5a, the CW laser started when the pump power reached 1 W. The CW output power increased linearly (the slope efficiency and optical conversion efficiency were 20.5% and 16.8%) with increasing pump power. The starting threshold of the PQS laser based on Ta_2_NiS_5_-SA was 2.5 W. At the pump power of 5.5 W, a maximum PQS laser output power of 0.275 W with optical conversion efficiency and slope efficiency of 5% and 9%was obtained. The difference in starting thresholds is due to the loss of saturable absorbers. And experiments were carried out with a power of less than 5.5 W to protect Ta_2_NiS_5_-SA from damage. The center wavelengths of the CW and PQS lasers were 1064.93 nm and 1065.17 nm, as displayed in Figure 5b. The relationships between the single pulse energy, peak power, and pump power are illustrated in Figure 5c. The maximum single pulse energy and peak power were 1.265 μJ and 7 W, respectively. The pulse duration and repetition rate versus pump power are shown in Figure 5d. The pulse width decreased (600–180 ns), but the repetition rate increased (166.7–217.4 kHz) with the increasing power (2.5–5.5 W). The pulse train and the single pulse profile at the highest pump power (5.5 W) are shown in Figure 6a. Figure 6b displays the beam profiles of the PQS laser at the pump power of 5.5 W; the output transverse modes of the PQS Nd: YAG laser is TEM_00_ mode, and the spot energy distribution presents a Gaussian distribution, which means that the output laser beams have high quality.

**Figure 7:**
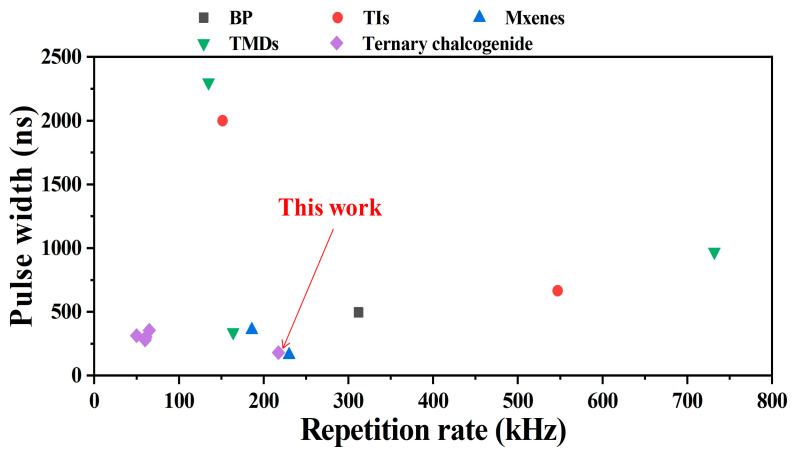
Pulse width versus repetition rate of PQS solid-state lasers based on different new 2D materials.

In laser application, short pulse width and high optical efficiency are both important to a PQS bulk laser [43]. Table 1 summarizes the laser performances of PQS solid-state lasers with different new 2D material-SAs. The corresponding data (pulse width versus repetition rate) are shown in Figure 7; the different marks represent different new 2D materials. Compared with the Ta_2_NiS_5_-SA applied in 1.9 μm PQS bulk laser [30], the 1 μm PQS bulk laser based on Ta_2_NiS_5_-SA in this letter shows a shorter pulse width (approximately 57.5%). The different optical efficiency may be due to the different linear absorption (1.9 μm: ~19%, 1.0 μm: ~27.4%) and nonlinear absorption properties (1.9 μm: ∆*T* = 12.2%, *I_sat_* = ~5.1 MW/cm^2^, NL = 6.9%, 1.0 μm: ∆*T* = 7.3%, *I_sat_* = ~0.32 GW/cm^2^, NL = 11.6%) of Ta_2_NiS_5_ materials in 1.9 μm [30] and 1 μm. But compared with new 2D material SAs applied at the same NIR region (1 μm), narrower pulse width and relatively high optical efficiency were both obtained based on Ta_2_NiS_5_-SA. Notably, an optimized cavity design and an excellent SA are essential to achieving a high-quality PQS laser [44]. During the experiments, the Ta_2_NiS_5_-SA possessed good characteristics, which could still maintain good modulation performance after placing in air for many days or after a long period of high-power excitation. Thus, the as-prepared Ta_2_NiS_5_ -SA would be a good candidate for PQS solid-state lasers in the NIR region.

### 3.2. Ultrafast Fiber Pulsed Lasers

#### 3.2.1. ML YDF Laser Operating at 1 μm

**Figure 8:**
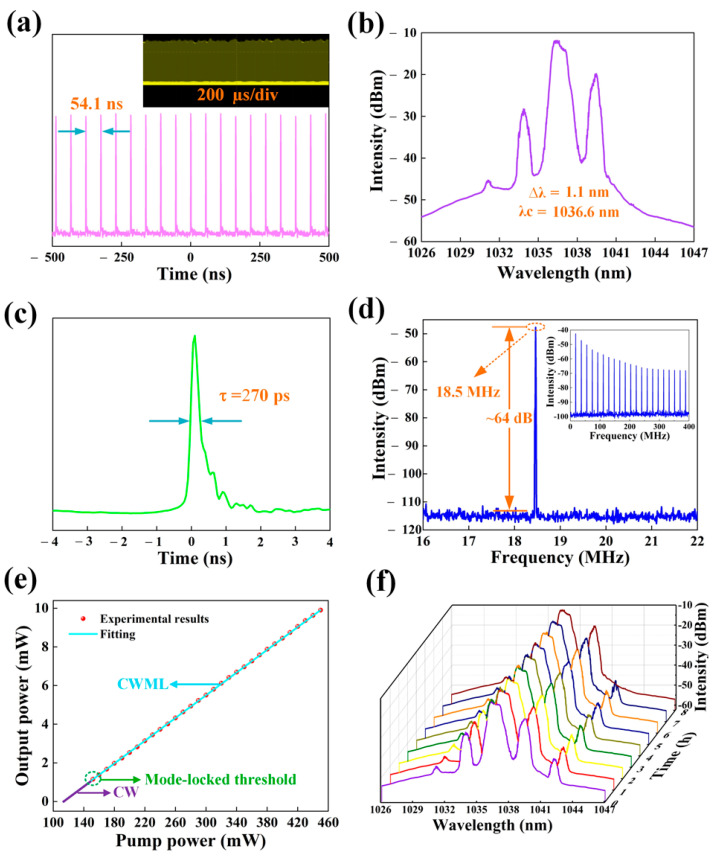
Typical ML YDF pulse characteristics. (**a**) Pulse train. (**b**) Optical spectrum. (**c**) Measurement of the laser pulse width. (**d**) RF spectrum (inset: wideband RF spectrum) of the ML pulses. (**e**) Variation in the output power with the pump power. (**f**) Optical spectra measurements at 1 h intervals over 8 h.

ML operation of the YDF laser was obtained when the pump power reached 155 mW by adjusting the PC in the cavity Figure 8 illustrates the characteristics of the ML fiber laser. Figure 8a shows the ML pulse train. The time interval between each pulse was 54.1 ns, which well matches the laser cavity length of 16.9 m. Figure 8b displays the optical spectrum of the ML pulses. The obtained central wavelength was located at 1036.6 nm, and the 3 dB spectral bandwidth was 1.1 nm. The trace of an ML laser pulse measured with the high-speed oscilloscope is shown in Figure 8c. The FWHM of the laser pulse duration was 270 ps. A strong signal peak with an ML repetition rate of 18.5 MHz was observed, and the signal-to-noise ratio (SNR) was measured to be approximately 64 dB, as displayed in Figure 8d, indicating that the obtained laser pulses have relatively high stability. The relationship between pump power and output power is recorded in Figure 8e, and the slanting efficiency is 2.7% by fitting a straight line. As shown in Figure 8f, the spectra were very stable with a small change within 8 h according to continuous monitoring of the output spectra of the YDF laser. In Table 2, we compared output characteristics of fiber lasers based on various 2D material SAs, and our results are similar to those based on other 2D materials.

#### 3.2.2. ML EDF Laser Operating at 1.5 μm

**Figure 9:**
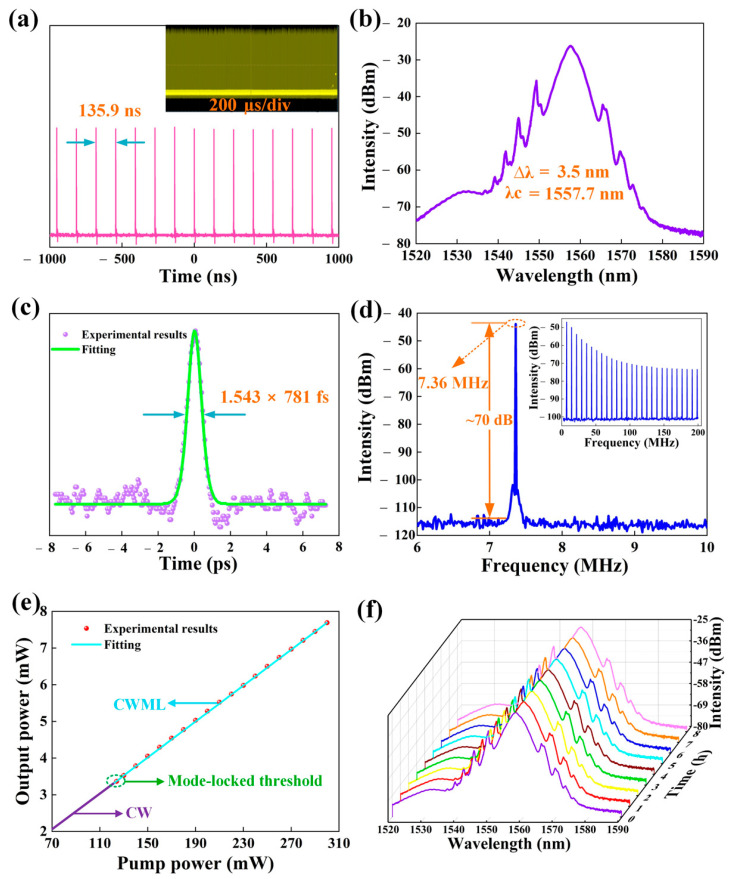
Typical ML EDF pulse characteristics. (**a**) Pulse train. (**b**) Optical spectrum. (**c**) Measurement of the laser pulse width. (**d**) RF spectrum (inset: wideband RF spectrum) of the ML pulses. (**e**) Variation in the output power with the pump power. (**f**) Optical spectra measurements at 1 h intervals over 8 h of operation.

To prove that Ta_2_NiS_5_ can work over a wider range of wavelengths, the fabricated Ta_2_NiS_5_-SA was used in the EDF laser cavity to generate an ultrafast laser pulse at approximately 1.5 μm. When the pump power reached 125 mW, stable ML laser pulses were observed by rotating the PC in the intracavity. Figure 9 shows the characteristics of the EDF ML laser. Figure 9a displays the time trace of the oscilloscope with an interval of 135 ns, corresponding to a repetition rate of 7.36 MHz. The illustration in Figure 9a illustrates the ML laser’s uniform intensity pulses, confirming the ML laser’s stability. As displayed in Figure 9b, the optical spectrum of the ML laser was centered at 1557.7 nm with a 3 dB spectral width of 3.5 nm. The autocorrelation trace for the soliton ML fiber laser is displayed in Figure 9c. The pulse autocorrelation trace’s full width at half maximum (FWHM) was 1.205 ps. The hyperbolic sech^2^ function is used to fit the autocorrelation trace curve measured in the experiment. Through the deconvolution factor of the sech^2^ pulse model of 1.543, the actual pulse width can be calculated as 781 fs. The calculated time-bandwidth product is 0.338.

**Figure 10:**
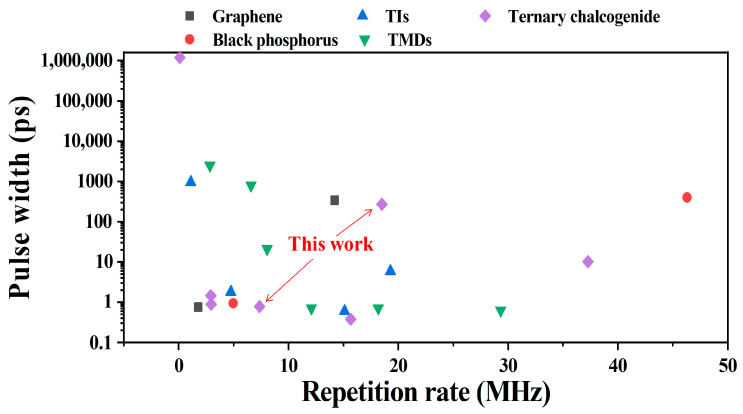
Pulse width versus repetition rate of PQS solid-state lasers based on different new 2D materials.

Table 2 presents an output performance comparison of ML fiber lasers based on various 2D material SAs, including graphene, BP, TIs, and TMDs. Figure 10 shows the corresponding pulse width and repetition rate. Notably, the pulse width in our results is approximately 85.6% shorter than those of other Ta_2_NiS_5_-SA fiber lasers. Figure 9d presents the radio frequency (RF) spectrum of ML pulses with a basic repetition rate of 7.36 MHz, consistent with the cavity length of 27.1 m. The SNR of the fundamental frequency was shown to be 70 dB, indicating a highly stable ML operation. The dependence between the average output power of the ML pulses and the pump power was measured, as shown in Figure 9e, with good linearity and a slope efficiency of 2.5%. The spectra were recorded every one hour. Figure 9f shows the optical spectral evolution of the pulse over 8 h, indicating the good stability of the ML EDF laser.

## 4. Conclusions

In summary, two kinds of high-quality Ta_2_NiS_5_-SA were successfully fabricated by the LPE method and applied in NIR bulk and fiber pulse lasers. The nonlinear absorption of the Ta_2_NiS_5_-SA was characterized by Z-scan and P-scan measurements at 1 μm and 1.5 μm, respectively. A 1 μm PQS bulk laser with a pulse width of 180 ns based on Ta_2_NiS_5_-SA was realized and demonstrated. A 70 dB signal-to-noise ML fiber laser based on Ta_2_NiS_5_-SA with evanescent field interactions was achieved at 1.5 μm with a pulse duration of 781 fs, which is shorter than the previous record for Ta_2_NiS_5_. Similarly, the output characteristics of the ML pulse in the YDF laser include a duration of 270 ps. The Ta_2_NiS_5_-SA made by the LPE method was applied in the ML fiber lasers (evanescent field interactions) and PQS bulk lasers in the NIR wavelength region. The results indicate that Ta_2_NiS_5_-SA prepared by the LPE method can be applied in 1 μm bulk PQS laser and improved by the new combination mode (evanescent field interactions) for better output performance of the fiber lasers.

## Figures and Tables

**Table 1:** Comparison of PQS solid-state lasers with different new 2D Materials-SAs.

Materials	Incorporation Method	SAs	λ (μm)	Pulse Width (ns)	Repetition Rate (KHz)	Optical Efficiency (%)	Ref
BP	ME	BP	1.0	495	312	3.7	[45]
TIs	HI/E	Bi_2_Te_3_	1.0	2000	151.5	3.2	[46]
		Bi_2_Se_3_	1.0	666	547	1.7	[47]
MXenes	LPE	Ti_3_C_2_T_x_	1.0	359	186	2.3	[48]
		Ti_2_CT_x_	1.0	163	230	21	[49]
TMDs	MS	WS_2_	1.0	2300	135	2.9	[50]
	PLD	MoS_2_	1.0	970	732	8.3	[51]
	CVD	PdSe_2_	1.0	340	164	4.6	[52]
Ternary chalcogenide	LPE	Ta_2_NiSe_5_	2.8	280	60	/	[53]
			2.0	302	61	/	
			1.0	355	65	/	
		Ta_2_NiS_5_	1.9	313	50	43.2	[30]
			1.0	180	217.4	5	This work

**Table 2:** Output performance comparison of ML fiber lasers based on various new 2D material-SAs.

Materials	Incorporation Method	SA	Pulse Width (ps)	Wavelength (nm)	Repetition Rate (MHz)	Pulse Energy (nJ)	SNR (dB)	Ref
Graphene	LPE	GR	340	1059.7	14.2	0.148	65	[54]
	CVD		0.756	1565	1.79	1.12	65	[9]
Black phosphorus	LPE	BP	400	1030.6	46.3	0.7	49	[55]
			0.94	1566.5	4.96	1.13	50	[12]
TIs	LPE	Bi_2_Te_3_	960	1064.47	1.11	1.08	60	[56]
	ME		0.6	1547	15.11	0.053	65	[41]
	MS	Sb_2_Te_3_	5.9	1047.1	19.28	0.21	71	[57]
	ME		1.8	1558.6	4.75	0.105	60	[21]
TMDs	LPE	WS_2_	2500	1030.3	2.84	2.82	48	[58]
			21.1	1565.5	8.05	0.22	NA	[59]
	HI/E	MoS_2_	800	1054.3	6.58	1.41	50	[51]
			0.71	1569.5	12.09	0.147	60	[15]
	LPE	NbS_2_	0.709	1559.36	18.18	1.28	NA	[17]
		SnS_2_	0.623	1562.01	29.33	0.041	45	[19]
Ternary chalcogenide	CVT	Mo_x_Ta_(1−x)_Se_2_	0.377	1532	15.659	0.672	/	[60]
		ReS_2(1−x)_Se_2x_	0.888	1561.15	2.95	0.275	53	[61]
	ME	Ta_2_NiS_5_	10.15	1029	37.27	1.017	62	[33]
			1.45	1569	2.92	6.37	67	
	LPE		1.2 × 10^6^ (PQS)	2803.7	0.1	1.64	42.4	[62]
			270	1036.6	18.5	0.535	64	This work
			0.781	1557.7	7.36	0.977	70	

## Data Availability

The data presented in this study are available on request from the corresponding author.
